# Oosorption in the Endoparasitoid, *Pteromalus puparum*

**DOI:** 10.1673/031.011.9001

**Published:** 2011-07-17

**Authors:** Jian-yang Guo, Sheng-zhang Dong, Gong-yin Ye, Kai Li, Jia-ying Zhu, Qi Fang, Cui Hu

**Affiliations:** ^1^ State Key Laboratory of Rice Biology, Ministry of Agriculture Key Laboratory of Molecular Biology of Crop Pathogens and Insects, Institute of Insect Sciences, Zhejiang University, Hangzhou, 310029, China; ^2^ Zhejiang Provincial Key Laboratory of Biometrology and Inspection and Quarantine, College of Life Science, China Jiliang University, Hangzhou 310018, China

**Keywords:** oocyte, starvation, ultrastructure, vitellin, vitellogenin

## Abstract

Oosorption is the resorption of oocytes in the ovaries, and is usually induced by environmental stress. It has been demonstrated in some insect species, but overall the mechanisms of oosorption are poorly understood. In this study, the oosorption in the endoparasitic wasp, *Pteromalus puparum* L. (Hymenoptera: Pteromalidae), was observed in response to starvation. To explore the details of oosorption in *P. puparum*, both levels of hemolymph vitellogenin and ovarian vitellin were determined using sandwich ELISA. The results indicated that both levels of vitellin and total protein in the ovaries were significantly decreased 48 h after eclosion in starved *P. puparum*, while those of vitellogenin and total protein in the hemolymph were increased. In addition, observation of the ultrastructure of mature oocytes in the ovarioles revealed changes in yolk protein content. Those protein yolk spheres and lipid yolk spheres that had accumulated in the oocytes, were transferred out of the oocytes of starved females. It was assumed that once oosorption was induced in *P. puparum*, vitellin in the oocytes was transported outside and released into the hemolymph. This information helps to elucidate a mechanism of oosorption in insects.

## Introduction

Oosorption, the resorption of oocytes in the ovary, can be induced by environmental stress such as starvation, mating delay, host deprivation, and the lack of available oviposition sites. This process has been demonstrated in various insect species (King and Ratcliffe 1969; Bell and Bohm 1975; Rivero-Lynch and Godfray 1997; Rosenheim et al. 2000; Kotaki 2003; Asplen and Byrne 2006; De Souza et al. 2007). Resorption of oocytes is assumed, in most cases, to be a way of recycling eggs that have reached maturity, but which have not been used in oviposition (Rivero and Godfray 1997). It may be considered to be an adaptive means of recruiting resources for future reproduction (Barrett et al. 2007). When oosorption is induced, eggs load in the ovary are decreased and apoptosis of the oocytes occurs, but the physiological mechanisms involved in this process have never been well elucidated (Papaj 2000; Rosenheim et al. 2000; Asplen and Byrne 2006).

In the previous studies, morphological characteristics of the ovary related to vitellogenesis have been well documented in insects. The uptake and transportation of yolk protein precursors, vitellogenins, by a receptor-mediated process during oogenesis are clearly understood (Raikhel and Dhadialla 1992; Raikhel 2005; Snigirevskaya and Raikhel 2005; Tufail and Takeda 2009). However, information on the resorption of ovaries remains fragmented. Both of these processes are critical to understanding the reproductive mechanisms of insects. From the previous studies, oosorption was characterized by reduction in total number of oocytes in the ovary and the appearance of space between oocytes (King and Ratcliffe 1969; Kotaki 2003), but only a few studies have been conducted on variations of oocyte ultrastructure during this process (Richards and King 1967; Asplen and Byrne 2006). Whether this phenomenon and the associated physiological mechanism commonly occur in insects, especially in parasitic wasps, has not been well reported (Kotaki 2003).

*Pteromalus puparum* L. (Hymenoptera: Pteromalidae) is a widely distributed endoparasitoid of papilionid and pieridid butterfly pupae, which plays an important role in integrated pest management (Hu 1983). In our previous studies, it is evident that vitellogenesis of *P. puparum* is initiated after the pupal stage and completed at the adult stage 48 h after eclosion (Dong et al. 2007), and adult nutrition significantly affects ovarian development (Dong et al. 2008). In addition, biochemical characterization of vitellogenin or vitellin (Dong et al. 2007), cloning of the vitellogenin gene (Ye et al. 2008), the process of vitellogenesis and its endocrine regulation (Dong et al. 2007, 2009), and oogenesis and programmed cell death of nurse cells (Dong et al. 2010) have been well studied. However, events and mechanisms during the following adult stage, especially when *P. puparum* face poor environmental conditions, are not known clearly.

Therefore, the studies reported here, using *P. puparum* as a model, are designed to provide detailed descriptions of oosorption in this parasitic wasp, verify whether starvation could induce oosorption or not, and describe the internal changes of oocytes during this process. The morphology of the ovary, the ultrastructure of oocytes in the basal part of the ovariole, which is connected with the ovarian calyx, and the vitellogenin/vitellin levels in hemolymph/ovaries of starved females are presented.

## Materials and Methods

### Insects

The colony of the endoparasitoid, *P. puparum*, was maintained in the laboratory on its host, *Pieris rapae* L. (Lepidoptera: Pieridae), at 25 ± 1° C and 70% relative humidity with a photoperiod of 14:10 L:D, as previously described by Cai et al. (2004). After eclosion, both female and male wasps were held together in glass jars (50 mm × 230 mm) to be presumed for mating, and fed with 20% (v/v) honey solution for at least 48 h. The female *P. puparumwasps* were then transferred individually to a glass tube (18 mm × 82 mm) to be allowed to lay eggs into newly-pupated host pupae of *Papilio xuthus* L. (Lepidoptera: Papilionidae), which generally occurs as an important pest attacking orange trees and is parasitized by *P. puparum* (Takagi ,1976; Guo et al. 2007). Extensive care was taken to make sure that each host received a single oviposition, and newly emerged female *P. puparum* were separated into two groups: one group starved until death, and the other fed with 20 % (v/v) honey solution. Under the same environmental conditions as described above, *P. puparum* were reared to various developmental stages or ages to be used in the following experiments.

### Ovary samples

The ovaries were dissected individually from female adults, starved or not, at different development stages ranging from 36 to 108 h after eclosion in phosphate-buffered saline (PBS) at pH 7.2 with protease inhibitor cocktail containing 0.1 M phenylmethyl sulfonyl fluoride (PMSF) (AMRESCO, www.amresco.com), 0.5 mg/ml leupeptin (Sigma), and 0.5 mg/ml aprotinin (Sigma, www.sigmaaldrich.com). Dissected ovaries were collected into a sterilized Eppendorf centrifuge tube embedded in ice, as described previously by Dong et al. (2007). Two pairs of ovaries per sample were then homogenized in an Eppendorf tube, and centrifuged at 10,000×g for 20 min at 4° C. Ten such ovary samples were prepared at each sampled stage. The supernatants were collected and stored at -70° C before being used to measure vitellin uptaken by the ovaries.

### Hemolymph samples

Female adult wasps were anaesthetized on ice, and hemolymph samples were prepared by carefully dissecting and opening the body cavity of two *P. puparum* per sample in 50 µ 1 of PBS with protease inhibitor cocktail as described above without breaking the ovaries or other organs, as described previously by Dong et al. (2007). The ages of adult females were the same as those for the sampled ovaries. Ten *P. puparum* were prepared at each sampled stage. For each sample, a drop of PBS with hemolymph was transferred to a sterilized Eppendorf tube on ice and centrifuged for 20 min at 10,000×g and 4° C to remove debris. These samples for measurement of vitellogenin levels were collected at different sampled stages and stored at -70° C before being detected by ELISA.

### Observation of ovarian morphological changes during oosorption

The ovaries were prepared as described above and carefully transferred to a clean concave slide containing 1% aceto-carmine buffer, as described by King and Richards (1968). The ovary samples dissected at different developmental stages were dyed with 1% aceto-carmine buffer (1 g aceto-carmine into 100 ml sterilized PBS) for 5 min. After washing with deionized water 3 times, 5 min per washing, samples were washed in solution containing 1 % HCl, and 70% ethanol for 15 min. The samples were then transferred to another slide containing deionized water. The morphology of ovary samples was photographed with a dissecting stereomicroscope (MZ16 A, Leica, www.leica-microsystems.com).

### Vitellogenin and vitellin determination

The vitellogenin levels in the hemolymph and vitellin levels in the ovaries of each sample were individually measured using indirect double antibody sandwich ELISA following the method established previously by Dong et al. (2007). Polyvinyl 96-well microplates (Nunc/Thermoscientific, www.thermoscientific.com) were, coated overnight at 4° C with a 1:5000 dilution of purified monoclonal antibody against *P. puparum* vitellin (100 µl/well). Then the microplates were washed three times with TBST (20mM Tris, pH 7.5; 150 mM NaCl; 0.05 % Tween-20). After incubation with 1 % bovine serum albumin for 30 min at 37° C, the hemolymph and ovarian samples (100 µl/well) were added into the coated plates and incubated for 1.5 h, followed by another three times washings with TBST. A dilution series of purified *P. puparum* vitellin (Dong et al. 2007) as the reference standard was used. Then, the purified rabbit antiserum against *P. puparum* vitellin (10 µg/ml) and the phosphatase-labeled goat anti-rabbit conjugate (Sigma) (1:10,000 dilution) were added separately to each well (100 µl/well) and incubated for 1 h. After three washings of TBST and another washing of TBS (20 mM Tris, pH 7.5 containing 150 mM NaCl), 100 µl aliquots of enzyme substrate *p*-nitrophenyl phosphate (Sigma) in 10% diethanolamineHCl (1 mg/ml, pH 9.8) were added. Colorimetric readings were recorded 30 min later with a Bio-Tek (www.biotek.com) ELISA reader at OD 450 nm.

### Total protein determination

The total protein in the hemolymph and ovaries was determined using a protein “quantization kit” (Applygen, www.applygen.com). The samples and a dilution series of bovine serum albumin (4 mg/ml) were added to the sterilized Eppendorf tubes. After mixing the samples and the binding solutions, the mixtures were kept at room temperature for 2 min. The solution was added to a 96-well microplate (100 µl/well). Colorimetric readings were recorded immediately at OD 595 nm.

### Transmission Electron Microscopy

Samples for examining the ultrastructure of resorptive oocytes or mature eggs were prepared by dissecting the ovaries of single adult females, which had been either fed or starved for 48, 60, 72, 84, 96, and 108 h. As previously described by Zhu et al. (2007), each ovary was dissected and single eggs, located at the basal part of each ovariole were transferred into the fixative (4% formaldehyde, 1% glutaraldehyde, with a phosphate buffer pH 7.2). The samples were cooled at 4° C for 30 min. This procedure was repeated twice per sample. The fixed samples were then kept at 4° C with excess fixation fluid for at least 12 h. Then, samples were rinsed three times for 45 min with PBS, 15 min per time.

Samples were post-fixed in 2% osmium tetroxide (OsO_4_) in deionized water at room temperature for 60 min. One 5-min rinse with deionized water at 4° C followed this procedure. The samples were dehydrated in sequentially increasing concentrations of alcohol (30, 50, 70, 95, and 100%) at room temperature, 15 min at each concentration. One 10-min and one 20-min rinse with 100% alcohol followed this procedure.

**Table 1.**
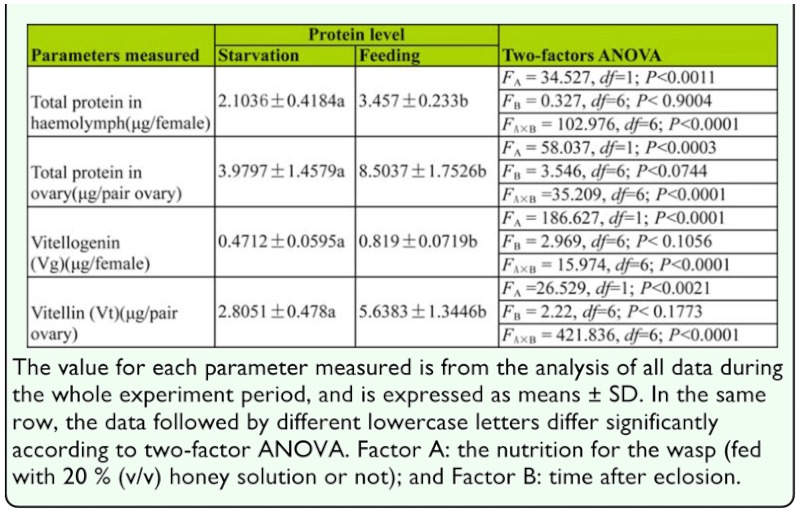
Two-factors ANOVA on protein level in *Pteromalus puparum* after eclosion.

All of the samples were immersed in 100% acetone in 1.5-ml centrifuge tubes for 20 min. The egg samples were then embedded in Epon 812. Ultrathin sections were double-stained with lead citrate and uranyl acetate, and examined using a JEX-1230 Transmission Electron Microscope (JEOL, www.jeol.com) at an accelerating voltage of 80 kV.

### Data analysis

All statistical analysis were conducted using the DPS© package (Version 8.01 for Windows) (Tang and Feng 2007). The effects of starvation and age on the levels of vitellogenin, vitellin, and total protein were analyzed using two-factor ANOVA and Tukey's multiple range test (MRT). To compare the difference in the levels of vitellogenin, vitellin, or total protein between starved and fed wasps, data were subjected to Student's *t*-test. All tests were considered significant at *P* < 0.05.

## Results

### Ovarian morphological changes during oosorption

**Figure 1.**
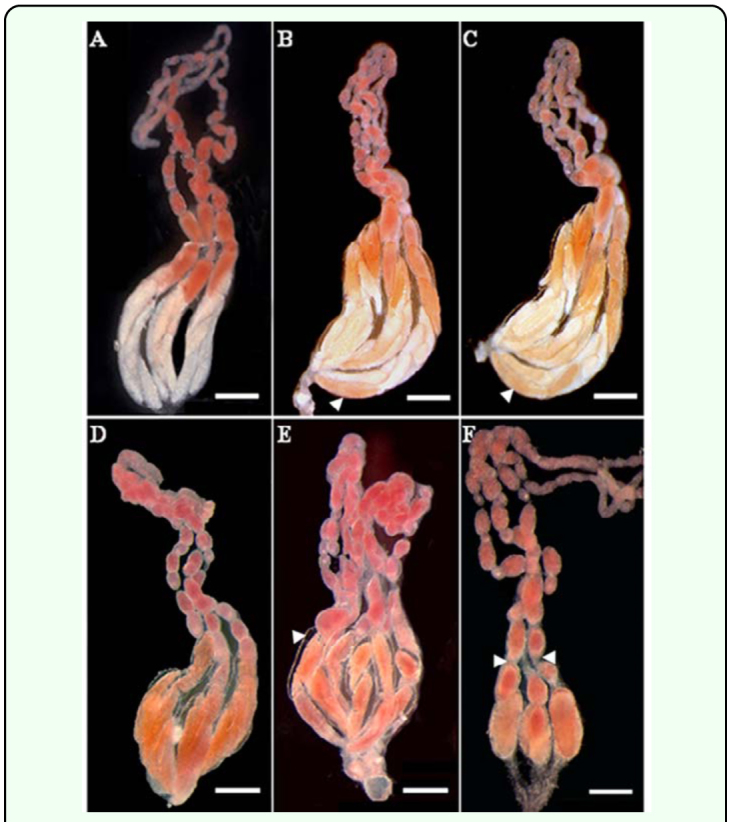
The morphology of ovaries stained with aceto-carmine solution in *Pteromalus puparum*. (A) Ovary of fed or starved adult 48 h after eclosion; (B) and (C) Ovaries of starved adult during 60–72 h after eclosion, salmon pink eggs present in the basal part of ovarioles (shown by the white arrows); (D–F) Ovaries of starved adult during 84–108 h after eclosion, more colored eggs, the spaces between eggs and the vacuoles were observed (shown by white arrows). Bars: A–F = 0.5 mm. High quality figures are available online.

The ovary of starved females presented distinctive characteristics when stained with aceto-carmine buffer (Figure 1). The ovaries presented similar morphology in fed adult females from 48 to 108 h after emergence (Figure 1A), which consisted of two diverse regions: the colorless mature egg region and the colored vitelarium region. The morphological characteristics of the ovary in starved females at 48 h were similar to those in the ovaries of fed females (Figure 1A) in several repetitions. However, in starved adults, some oocytes in the mature egg region were stained salmon pink 60 h after eclosion (Figure 1B, C), and some oocytes at the basal part of ovarioles were stained red 84–108 h after eclosion (Figure 1D–F).

Morphological alterations of ovaries were observed in 4-day-old starved females. The ovarioles showed alterations in the mature egg region, while the oocytes showed large vacuoles from 96 to 108 h (Figure 1D–F). Oocytes in this region were stained intensely at 84 h (Figure 1D) and there was a rapid reduction in size and number of the oocytes, especially mature oocytes from 96 to 108 h (Figure 1E, F). Spaces between mature eggs and the wall of ovarioles were also observed during this period (Figure 1F). However, oocytes in vitelarium region did not show significant changes in this experiment.

**Figure 2.**
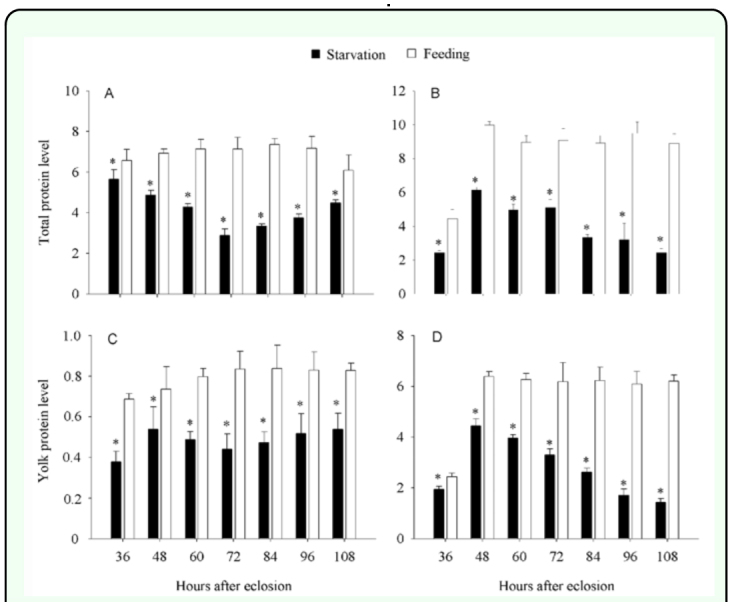
Differences in the levels of total protein and yolk proteins in hemolymph and ovaries between starved and fed *Pteromalus puparum* adults. (A) Total protein in hemolymph, µg/female; (B) Total protein in ovaries, µg/pair ovary; (C) Vitellogenin, vitellogenin, in hemolymph, µg/female; (D) Vitellin, vitellin, in ovaries, µg/pair ovary. (A–D) Feeding: adults fed on 20 *%* honey solution; Starvation: adults starved for days until death. The data are expressed as means ± standard derivation. At the same stage, values followed by the asterisk (*) represent significant differences (*P* < 0.05, Student's *t-*test). High quality figures are available online.

### Variations in both levels of yolk protein and total protein during oosorption

The levels of total protein in hemolymph and ovaries were examined using the Bradford method (Table 1; Figure 2A, B). Starvation during the adult stage had a significant effect on total protein levels in the hemolymph and ovary, although this effect varied with the age of tested *P. puparum* (Table 1). By comparison with fed adults at the same development stage, the levels of total protein both in hemolymph and ovaries of starved adults were significantly lower (Figure 2A, B). In fed adults, the maximum levels of total protein in hemolymph and ovaries were 3.6 µg/female 72 h after eclosion and 9.9 µg/pair ovary 48 h after eclosion, respectively. However, protein levels were lower in the starved adult *P. puparum* at 2.4 µg/female and 6 µg/pair ovary, respectively. In starved adults, an increased level of total protein in hemolymph 84 h after eclosion and a significantly decreased level of total protein in ovaries 60 h after eclosion were observed.

Using the indirect double antibody sandwich ELISA, changes in vitellogenin and vitellin levels in the hemolymph and ovary were determined after eclosion (Table 1; Figure 2C, D). The starvation had a significant effect on both levels of vitellogenin and vitellin, although the effect varied with age (Table 1). By comparison with fed adults at the same development stage, the level of vitellogenin in hemolymph for starved adults was significantly lower (Figure 2C). In starved adults, the levels of vitellogenin gradually decreased from 48 to 72 h after eclosion, and then slowly increased until 108 h after eclosion (Figure 2C). However, the levels of vitellogenin in fed adults markedly increased from 36 to 72 h after eclosion, and then almost kept a plateau. As regard to the levels of vitellin, starved adults were also significantly lower than fed adults as compared at the same developmental stage (Figure 2D). For both fed and starved adults, the levels of vitellin increased during the first 48 h after eclosion. After this, the levels of vitellin for starved adults showed a significant decrease until 108 h after eclosion, while those for fed adults almost remained highly constant.

**Figure 3.**
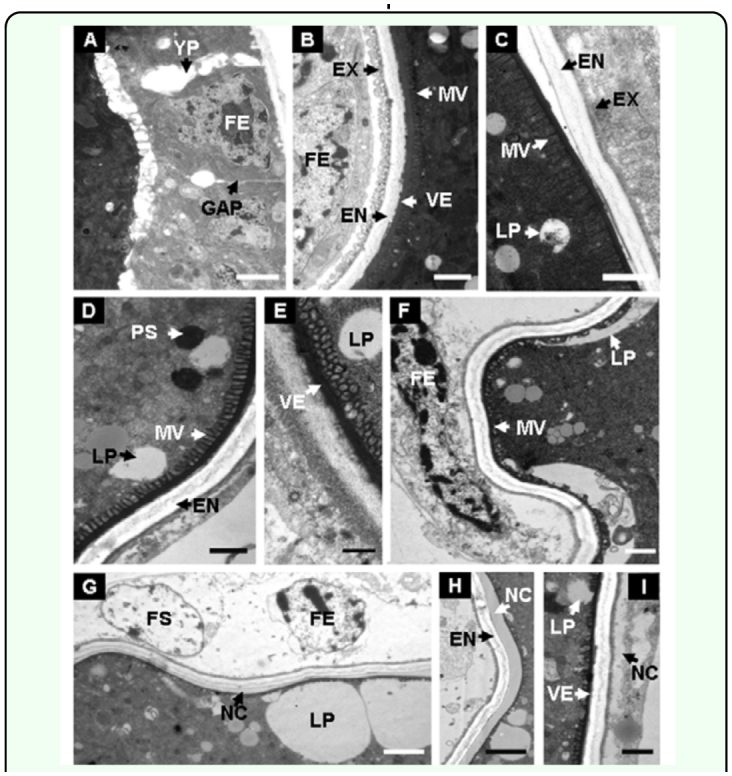
Ultrastructural analysis of oocytes in the basal part of ovariole. (A) Eggshell structure of fed or starved adult 24 h after eclosion, showing the gaps (arrows) in the cells of the follicular epithelium, FE, permit passage of yolk proteins to vitellogenic oocytes; (B–C) Intact eggshell in fed or starved adult 48 h after eclosion; (D, E) Details of the chorion 72 h after eclosion, showing the transportation of LPS; (F, G) Follicular cells break up; (H, I) New content around the eggshell was observed at the later stages. **PS**, protein yolk spheres; **LP**, lipid yolk spheres; **FE**, follicular cell; **MV**, microvilli; **VE**, vitelline envelope; **EN**, endochorion; **EX**, exochorion; **FS**, follicular sac; **NC**, New content. Bars: A, G, H = 2 µm; B, C, D, F, 1 = 1 µm E = 0.5 µm. High quality figures are available online.

### Ultrastructural analysis of reabsorbed oocytes

The ultrastructure of mature eggs in the basal part of ovarioles showed the details of oosorption (Figure 3). Gaps (arrows) were observed in the cells of the follicular epithelium around the developing oocytes 24 h after eclosion (Figure 3A), which permitted the passage of yolk contents to oocytes during oogenesis. The eggshell remained intact, and the follicular epithelium was well-arranged around the eggshell in fed adults and starved adults 48 h after eclosion. The eggshell, composed of vitelline envelope, endochorion, and exochorion (Figure 3B, C), was smooth and regular at the early stages of oosorption.

**Figure 4.**
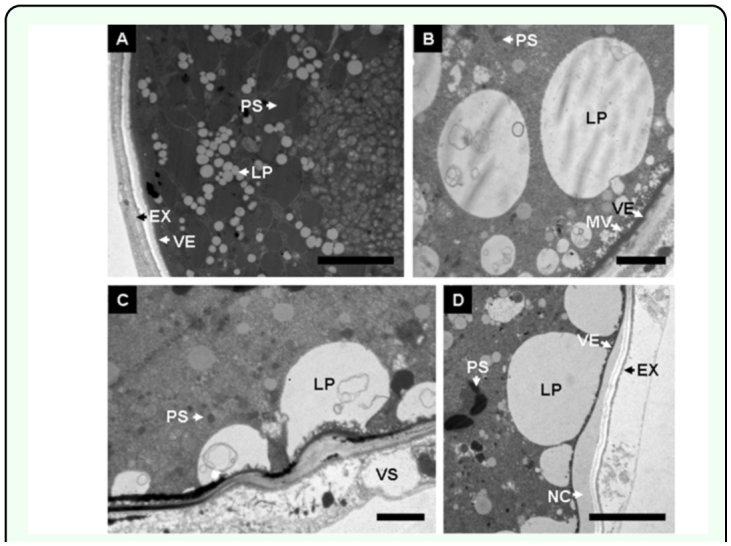
Transportation of yolk contents during oosorption. (A) Yolk content in egg of fed or starved adult 48 h after eclosion, showing the protein yolk spheres, lipid yolk spheres oocytes; (B–C) The transportation of yolk materials during 60–84 h after eclosion; (D) Details of the new eggshell formed at the later stages. **PS**, protein yolk spheres; **LP**, lipid yolk spheres; **FE**, follicular cell; **MV**, microvilli; **VE**, vitelline envelope; **EN**, endochorion; **EX**, exochorion; **FS**, follicular sac; **NC**, New content; **VS**, vesicle. Bars: A, D = 5 µm; B, C = 2 µm. High quality figures are available online.

Microvilli were observed in the internal part of eggshell (Figure 3D, E). However, the eggshell was severely twisted (Figure 3F) and the breakup of exochorion was observed at the later stage (Figure 3G). The follicular cells, surrounded the oocytes regularly at the early stage (Figure 3A, B), were transferred into follicular sac, or broken into pieces at the later stage (Figure 3F, G). New contents were arranged around the endochorion or out of the eggshell (Figure 3H, I).

Changes in eggshell structure were accompanied by the transportation of yolk sphere contents. The yolk spheres were composed of several shapes: white lipid yolk spheres, gray protein yolk spheres, and other types of spheres consisting of archiblast (Figure 4A). Visible lipid yolk spheres joined together and formed larger and amorphous spheres near the eggshell at 60 h after eclosion (Figure 4B). Liquid yolk spheres, which bound onto the small microvilli, permeated into the eggshell gradually and were then transferred out of exochorion and formed vesicles (Figure 4C). In other case, the yolk spheres were observed to form a layer of membrane composed of new contents after starvation for 84 h (Figure 4D). By contrast, most of the yolk spheres were observed to break down into small spheres or appeared to have formed irregular drops (Figure 4C, D).

## Discussion

Oosorption is an adaptive strategy in which the oocytes degenerate by interrupting yolk uptake in response to stress from behavioral, ecological, or physiological factors (Bell and Bohm 1975). Nutritional deficiency is the main cause of oosorption, but other factors such as season, parasitism, age, social pressure (reproductive dominance by an individual), and absence of mating are also closely associated with oosorption (King and Richards 1968; Bell and Bohm 1975; Patricio and Cruz-Landim 2002; De Souza et al. 2007). The present investigation shows that starvation, which leads to degeneration of mature oocytes, is an important factor inducing oosorption in *P. puparum*. This is in agreement with other reported results, for example in *Plautia crossota stali*, starvation experienced by young females was one of the factors inducing oosorption (Kotaki 2003). Oosorption in the cockroach *Leucophaea maderae* was induced when the adult females were starved for several days (Engelmann and Mala 2005). Structural variations revealed the presence of oosorption in host-deprived *Eretmocerus eremicus* (Asplen and Byrne 2006). Egg resorption at the basal part of ovariole was observed in the starved *Nasonia vitripennis* (King and Richards 1968). It is not clear whether oosorption is universal in insects, but is likely to be common.

In this study, it was found that *P. puparum* females released vitellin from the ovary into hemolymph when oosorption was induced by starvation, whereas this characteristic was not observed in fed females at the same stages. As far as we know, this is the first report to verify the transportation of yolk contents during oosorption. Older age combined with starvation further enhanced the resorption of nutritional contents in mature oocytes of *P. puparum*. The released contents from oocytes perhaps provide nutrition and energy for the survival of starved females. The decreased total protein levels of the ovary and increased total protein levels of hemolymph were observed in starved females 72 h after eclosion. Similar phenomena were also found in *Aphytis aonidiae*, where the resorption of immature eggs could reduce the cost of producing a mature egg (Rosenheim et al. 2000). We assumed that this may be a potential mechanism, allowing the female *P. puparum* increased benefits (prolonged life) and reduced costs (decreased egg load), to adopt variable environments.

The ultrastructure of eggs in the basal part of ovarioles was aimed to observe specific changes during oosorption. Ultrastructure of oocytes during oosorption had been described in detail only for other two parasitoid wasps, the *Nasonia vitripennis* (Richards and King 1967; King and Richards 1968) and *Eretmocerus eremicus* (Asplen and Byrne 2006). Oosorption by these wasps was induced by starvation or by other factors, and induced variation of the egg structure or yolk contents changed during the phosphatase. In *N. vitripennis* females, the oocytes in the basal part of ovarioles undergo resorption first (King and Richards 1968). This phenomenon can also be observed in *P. puparum* (Figure 1B, C). In *P. puparum*, the lipid yolk spheres accumulated, and then were transferred across the vitellin envelope, or perhaps together with the protein yolk spheres, were transferred across the exochorion by vesicles. It is possible that the assembled lipid yolk spheres and the fragmented degenerated protein yolk spheres were transported through the space of degenerated follicular sac into the hemolymph. This phenomenon was not observed in normal fed *P. puparum*.

## References

[bibr01] Asplen MK, Byrne DN (2006). Quantification and ultrastructure of oosorption in *Eretmocerus eremicus* (Hymenoptera: Aphelinidae).. *Journal of Morphology*.

[bibr02] Barrett EL, Preziosi RF, Moore AJ, Moore PJ (2007). Effects of mating delay and nutritional signals on resource recycling in a cyclically breeding cockroach.. *Journal of Insect Physiology*.

[bibr03] Bell WJ, Bohm MK (1975). Oosorption in insects.. *Biological Reviews of the Cambridge Philosophical Society*.

[bibr04] Cai J, Ye GY, Hu C (2004). Parasitism of *Pieris rapae* (Lepidoptera: Pieridae) by a pupal endoparasitoid, *Pteromalus puparum* (Hymenoptera: Pteromalidae): effects of parasitization and venom on host hemocytes.. *Journal of Insect Physiology*.

[bibr05] De Souza EA, Neves CA, de Oliveira Campos LA, Zanuncio JC, Serrão JE (2007). Effect of mating delay on the ovary of *Melipona quadrifasciata anthidioides* (Hymenoptera: Apidae) queens.. *Micron*.

[bibr06] Dong SZ, Ye GY, Hu C (2009). Roles of ecdysteroid and juvenile hormone in vitellogenesis in an endoparasitic wasp, *Pteromalus puparum* (Hymenoptera: Pteromalidae).. *General and Comparative Endocrinology*.

[bibr07] Dong SZ, Ye GY, Guo JY, Yu XP, Hu C (2010). Oogenesis and programmed cell death of nurse cells in the endoparasitoid, *Pteromalus puparum*.. *Microscopy Research and Technique*.

[bibr08] Dong SZ, Guo JY, Ye GY, Hu C (2008). Effect of adult nutrition and mating on ovarian development and vitellogenesis in the endoparasitoid, *Pteromalus puparum* (Hymenoptera: Pteromalidae).. *Acta Phytophylacica Sinica*.

[bibr09] Dong SZ, Ye GY, Zhu JY, Chen ZX, Hu C, Liu SS (2007). Vitellin of *Pteromalus puparum* (Hymenoptera: Pteromalidae), a pupal endoparasitoid of *Pieris rapae* (Lepidoptera: Pieridae): Biochemical characterization, temporal patterns of production and degradation.. *Journal of Insect Physiology*.

[bibr10] Engelmann F, Mala J (2005). The cockroach *Leucophaea maderae* needs more than juvenile hormone, vitellogenin and reserves to make a yolky egg.. *Journal of Insect Physiology*.

[bibr11] Guo JY, Dong SZ, Ye GY, Liu XQ (2007). Comparison of ovarian development in *Pteromalus puparum* from its two host *Papilio xuthus* and *Pieris rapae*.. *Journal of Environmental Entomology*.

[bibr12] Hu C (1983). A survey on the parasites of the small white butterfly, *Artogeia rapae* (L.) in China.. *Acta Entomologica Sinica*.

[bibr13] King PE, Ratcliffe NA (1968). Oosorption in *Nasonia vitripennis* (Hymenoptera: Pteromalidae).. *Journal of Zoology*.

[bibr14] King PE, Ratcliffe NA (1969). Structure and possible mode of function of the female reproductive system in *Nasonia vitripennis* (Hymenoptera: Pteromalidae).. *Journal of Zoology*.

[bibr15] Kotaki T (2003). Oosorption in the stink bug, *Plautia crossota stali*: induction and vitellogenin dynamics.. *Journal of Insect Physiology*.

[bibr16] Papaj DR (2000). Ovarian dynamics and host use.. *Annual Reviews of Entomology*.

[bibr17] Patricio K, Cruz-Landim C (2002). Mating influence in the ovary differentiation in adult queens of *Apis mellifera* L. (Hymenoptera, Apidae).. *Brazilian Journal of Biology*.

[bibr18] Raikhel AS, Brown MR, Bellés X, Gilbert LI, Iatrou K, Gill SS (2005). Hormonal control of reproductive processes.. *Comprehensive Molecular Insect Science*.

[bibr19] Raikhel AS, Marquardt WC (2005). Vitellogenesis of disease vectors, from physiology to genes.. *Biology of Disease Vectors*.

[bibr20] Richards JG, King PE (1967). Chorion and vitelline membranes and their role in resorbing eggs of the hymenoptera.. *Nature*.

[bibr21] Rivero-lynch AP, Godfray HCJ (1997). The dynamic of egg production, and resorption in a parasitoid wasp.. *Functional Ecology*.

[bibr22] Rosenheim JA, Heimpel GE, Mangel M (2000). Egg maturation, egg resorption and the costliness of transient egg limitation in insects.. *Proceedings of the Royal Society B: Biological Sciences*.

[bibr23] Takagi M (1976). Ecology of *Pteromalus puparum* (Linne) (Hymenoptera: Pteromalidae), parasitic on the pupae of *Papilio xuthus* Linne (Lepidoptera: Papilionidae).. *Japanese Journal of Applied Entomology and Zoology*.

[bibr24] Tang QY, Feng MG (2007). *DPS Data Processing System: Experimental Design, Statistical Analysis and Data Mining*..

[bibr25] Tufail M, Takeda M (2009). Insect vitellogenin/lipophorin receptors: molecular structures, role in oogenesis, and regulatory mechanisms.. *Journal of Insect Physiology*.

[bibr26] Ye GY, Dong SZ, Song QS, Shi M, Chen XX, Hu C (2008). Molecular cloning and developmental expression of the vitellogenin gene in the endoparasitoid, *Pteromalus puparum*.. *Insect Molecular and Biology*.

[bibr27] Zhu JY, Ye GY, Hu C (2007). Morphology and ultrastructure of the venom apparatus in the endoparasitic wasp *Pteromalus puparum* (Hymenoptera: Pteromalidae).. *Micron*.

